# Does family history of metabolic syndrome affect the metabolic profile phenotype in young healthy individuals?

**DOI:** 10.1186/1758-5996-6-75

**Published:** 2014-06-26

**Authors:** Anna Lipińska, Magdalena Koczaj-Bremer, Krzysztof Jankowski, Agnieszka Kaźmierczak, Michał Ciurzyński, Aisha Ou-Pokrzewińska, Ewelina Mikocka, Zbigniew Lewandowski, Urszula Demkow, Piotr Pruszczyk

**Affiliations:** 1 Department of Internal Medicine and Cardiology, Medical University of Warsaw, Lindley’a 4, 02-005 Warsaw, Poland; 2 Department of Epidemiology, Medical University of Warsaw, Oczki 3, 02-007 Warsaw, Poland; 3 Department of Laboratory Diagnostics and Clinical Immunology of Developmental Age, Medical University of Warsaw, Marszałkowska 24, 00-567 Warsaw, Poland

**Keywords:** Metabolic syndrome, Family history, Cardiovascular disease, Risk factors

## Abstract

**Background:**

Early identification of high-risk individuals is key for the prevention of cardiovascular disease (CVD). The aim of this study was to assess the potential impact of a family history of metabolic syndrome (fhMetS) on the risk of metabolic disorders (abnormal body mass, lipid profile, glucose metabolism, insulin resistance, and blood pressure) in healthy young individuals.

**Methods:**

We studied CVD risk factors in 90 healthy volunteers, aged 27–39 years; of these, 78 had fhMetS and 12 were without fhMetS (control group). Fasting serum lipids, glucose, and insulin levels were assayed, and anthropometric parameters and blood pressure using, an ambulatory blood pressure monitoring system, were measured. Nutritional and physical activity habits were assessed.

**Results:**

Despite similar nutritional and physical activity habits, abnormal body mass was found in 53.2% of the fhMetS participants and 46.1% of the control participants (p = 0.54). The occurrence of obesity was 19.4% and 0%, respectively (p = 0.69). Compared to the control participants, fhMetS was associated with significantly higher total cholesterol (5.46 mmol/L vs. 4.69 mmol/L, p < 0.030), low-density lipoprotein cholesterol ( 3.28 mmol/L vs. 2.90 mmol/L, p < 0.032), and non-high-density lipoprotein cholesterol ( 3.74 mmol/L vs. 3.25 mmol/L, p < 0.016) levels, in addition to lower fasting glucose levels ( 4.51 mmol/L vs. 4.81 mmol/L, p < 0.042). A positive correlation between fasting glucose and insulin levels (r = 0.28; p < 0.015) was detected in the fhMetS participants. Higher mean daytime systolic blood pressure (121.5 mmHg vs. 113.3 mmHg, p < 0.035), mean daytime diastolic blood pressure ( 79.0 mmHg vs. 74.5 mmHg, p < 0.045), and mean nighttime diastolic blood pressure ( 64.0 mmHg vs. 59.5 mmHg, p < 0.019) were observed in the fhMetS group.

**Conclusions:**

More than 50% of the fhMetS participants had excess weight or a lipid disorder, which may indicate an increased risk of cardiovascular disease and the need for regular ambulatory assessment of serum lipid concentrations in young people with a family history of MetS.

## Background

Atherosclerotic cardiovascular disease (CVD) continues to be a major cause of premature mortality worldwide and is considered by the Wold Health Organization (WHO) to be a major chronic, noncommunicable disease that results in global socioeconomic burden. Owing to the increasing prevalence of risk factors, the incidence of CVD will also continue to increase [1]. The prevalence of atherosclerosis, even in early life, in addition to its dynamic course and unfavorable long-term prognosis, especially in young people, requires novel, easy, accessible, and more aggressive action in primary prevention. The WHO estimates that effective preventive strategies targeted at established, easy to assess, and modifiable risk factors such as smoking, obesity, dyslipidemia, diabetes, and arterial hypertension have the potential to reduce the global cardiovascular disease mortality rate by as much as 80% [2,3]. However, the fundamental issue remains how to effectively identify groups of patients with increased cardiovascular risk, especially among the young and healthy. Several studies have shown that individuals with a family history of CVD are such a group [4]. The current 2012 European Guidelines on cardiovascular disease prevention in clinical practice note an increased cardiovascular risk in individuals with a family history of premature CVD. At the same time, the Systematic COronary Risk Evaluation (SCORE) system includes a general approach to family history, which tends to lower the associated cardiovascular risk [5].

Metabolic syndrome (MetS), includes several particularly unfavorable, proatherogenic, and interacting metabolic disturbances (visceral obesity, hyperinsulinemia, insulin resistance, elevated serum glucose, dyslipidemia, and arterial hypertension) and is a major cause of atherosclerotic cardiovascular disease and type 2 diabetes [6]. The presence of 3 or more of the MetS components increased the risk of cardiovascular disease 3–5 fold [7]. The incidence of MetS is also increasing, and studies indicate that the current incidence is 10- 30% in various populations worldwide [7-10]. Studies have demonstrated a strong genetic basis for MetS components; therefore, young people with a family history of MetS could be regarded to be at higher cardiovascular risk and included in early preventive efforts to reduce those risk factors. However, evidence regarding the best methods to identify those at higher risk is lacking. We hypothesized that apparently healthy offspring of parents with MetS may demonstrate metabolic alterations that are typical for MetS. Owing to the increased prevalence of MetS in the general population, it is likely that clinicians will encounter increasing numbers of young patients with a family history of MetS. To the best of our knowledge, this population has not yet been analyzed. Therefore, the aim of this study was to characterize young, clinically healthy adults with a family history of MetS and assess the typical features of MetS (abnormal body mass, lipid and carbohydrate metabolic disorders, insulin resistance, and arterial hypertension) in this same group.

## Material and methods

### Subjects and study design

The study sample consisted of 90 healthy subjects (50 women, 40 men) aged 27 – 39 years with no clinical signs of atherosclerosis. To identify the subjects for the study group, we asked approximately 2000 patients from our Diabetic and Cardiac Outpatient clinics to refer their healthy adult children to our study. From the group of 211 patients who agreed to refer their children, we selected 160 patients with definitive MetS, and we screened their 150 children. Of these, 78 healthy adults offspring of the parents with confirmed MetS were enrolled in the study and considered the family history of metabolic syndrome (fhMetS) group. The control group included 12 persons with no family history of MetS who were selected from a group of 20 subjects who matched the demographic characteristics of the study group with a special focus on nutritional habits and physical activity. The exclusion criteria included: symptomatic atherosclerosis, i.e. coronary artery disease, arterial occlusive disease, or arterial hypertension; history of stroke or TIA; heart defect, heart failure, diabetes, thyroid disease, acute or chronic inflammatory process, connective tissue disorder, allergic disease, kidney disease (estimated glomerular filtration rate < 60 ml/min), neoplasm, drug- or alcohol-dependency, smoking, obstructive sleep apnea syndrome, or visceral obesity.

The project was conducted with approval from the Ethics Committee of Medical University of Warsaw. The participants provided written informed consent prior to participation.

MetS diagnosis was determined based on data in medical records (anthropometric data, biochemical data, and medical history). MetS was defined according to the Third Report of the National Cholesterol Education Program adult treatment panel III criteria of 2004, and required ≥ 3 of the 5 equally weighted criteria: (1) waist circumference > 102 cm in men and > 88 cm in women, (2) blood pressure > 130/85 mmHg, or diagnosed and treated hypertension, (3) serum high density lipoprotein cholesterol (HDL-C) < 40 mg/dL (1.03 mmol/L) in men and <50 mg/dL (1.3 mmol/L) in women, (4) serum triglycerides (TG) ≥ 150 mg/dL (1.7 mmol/L) or diagnosed and treated dyslipidemia, or (5) fasting serum glucose ≥ 100 mg/dL (5.6 mmol/L) or diagnosed and treated diabetes [11]. The same criterion for waist circumference (WC) for Caucasians was defined in the 2009 scientific consensus [12].

All participants underwent an anthropometric assessment, which included measurements of WC, hip circumference, and weight. WC (cm) was measured as the midpoint between the lowest rib and the iliac crest. The waist-hip ratio (WHR), and body mass index (BMI) were calculated. BMI, WHR, and WC are all established, simple clinical tests to assess excess body mass. BMI is the most common indicator used in clinical practice and epidemiological studies owing to its repeatability and its confirmed correlation with WC [13,14]. Abnormal body mass in the present study was defined as BMI ≥25 kg/m^2^ and obesity as BMI ≥30 kg/m^2^.

### Biochemical measurements

Venous blood was collected in the morning, after fasting for at least 8 hours. All blood analyses were performed in the central laboratory of our hospital using Roche Diagnostic commercial kits and multichannel automatic analyzer Roche Cobas 6000 (c501 and e601). Fasting plasma glucose was measured using the enzymatic colorimetric hexokinase method (coefficient of variation [CV] less than 1.3%). Total plasma cholesterol (TC) and TG were assayed using the enzymatic colorimetric method with cholesterolesterase oxidase and glycerol phosphate oxidase, respectively (CV less 1.6% for TC and less than 2.0% for TG). HDL-C was assayed using the enzymatic colorimetric method with polyethylene glycol (CV less than 1.5%). Serum creatinine levels were assayed using the Jaffe method ( CV less than 3.5%). Aspartate transaminase (AST) and alanine aminotransferase (ALT) were assayed using the International Federation of Clinical Chemistry method (CV less than 3.1% for AST and 1.4% for ALT). Uric acid was assayed using the enzymatic colorimetric method with uricase (CV less than 1.6%). Glycated hemoglobin (HbA1c) was measured using a turbidimetric inhibition immunoassay (CV less than 2.0%). High sensitivity C-reactive protein (hsCRP) was measured using an immunoturbidimetric latex method (CV less than 1.3%). Low-density lipoprotein cholesterol (LDL-C) was calculated using the Friedewald formula (there were no subjects with TG > 400 mg/dL). Non-HDL-C was calculated using the following formula: TC– HDL-C = non-HDL-C. The insulin concentration was measured using an electrochemical method (Roche, ELECSYS 2010 syste), and the normal range is 2.5 – 24.9 μU/mL. Insulin resistance was assessed by homeostasis model assessment-estimated insulin resistance (HOMA- IR), which was calculated using the following formula: fasting insulin (mU/L) × fasting glucose (mmol/L) / 22.5 [15].

### Blood pressure measurements

Ambulatory blood pressure monitoring (24-hour ABPM) was performed using the Spacelabs ABPM system every 20 minutes during the day (6 am–10 pm) and every 30 minutes at night (10 pm–6 am). The mean daytime systolic blood pressure (D-SBP), mean daytime diastolic blood pressure (D-DBP), mean nighttime systolic blood pressure (N-SBP), and mean nighttime diastolic blood pressure (N-DBP) were calculated. A resting electrocardiogram was performed using 12 standard leads and the Spacelabs device, and a treadmill test was conducted (Cambridge Hart CH 2000, Cardiac Diagnostic System USA). Echocardiograms were performed using a Phillips IE 33 system (Andeouver, Massachusetts, USA) with a 2.5 – 3.5 MHz transducer.

### Nutritional habits and physical activity assessment

All subjects completed standardized questionnaires regarding nutritional and physical activity habits [16,17]. Questions for nutritional habits included the number of meals per day, and their timing; frequency and amount of vegetables, fruits, sweets, and meat; and type and quantity of fluids consumed. Physical activity was classified as low (sedentary life-style), if physical exercises lasting at least 30 minutes was performed, (not at all, once a week, several times a month, or less frequently). The moderate classification was defined as physical exercises 2–3 times per week. The high classification was defined as ≥ 4 times per week.

### Statistical analysis

Data are presented as median and interquartile range. Statistical analysis was performed using the one-tailed Mann–Whitney test for comparison of quantitative variables between 2 groups. Association between quantitative variables were tested using Spearman correlation coefficients. All analyses were conducted using SAS 9.3 (SAS Institute, Cary, NC, USA).

## Results

No differences were found between the groups in nutritional habits or level of physical activity. Approximately 20% of the subjects were moderately physically active, and none of the subjects participated in a high level of physical activity. There were no differences between the groups in age, weight, WC, BMI, or WHR, nor were there differences in the biochemical parameters (creatinine, uric acid, AST, ALT, erythrocyte sedimentation rate, white blood cell count, or hsCRP), which were within the normal ranges in both groups (Table 1). However, abnormal body mass, defined as BMI ≥ 25 kg/m^2^, was present in 53.2% of the fhMetS participants and 41.6% of the control participants. Obesity (BMI ≥ 30 kg/m^2^) was noted in 19.4% of the fhMetS participants and none of the control group. The fhMetS group had significantly higher total cholesterol (p < 0.030), LDL-C (p < 0.032), and non-HDL-C (p < 0.016) values than the control group (Table 2), with 67.5% and 59.7% of the fhMetS participants and 41.6% and 33.3% of the control participants with a TC concentration ≥ 4.94 mmol/L and a LDL-C concentration ≥ 2.99 mmol/L respectively. In addition the fhMetS group was characterized by a higher frequency of participants with a TG concentration ≥ 1.71 mmol/L than the control group (18.18% vs. 16.7%, p = 0.99), and a lower frequency of participants with an HDL-C concentration ≥ 1.30 mmol/L (62.3% vs. 66.6%, p = 0.99). The TG concentration was significantly higher (p < 0.01) and HDL-C levels were significantly lower (p < 0.001) in the fhMetS participants with an abnormal body mass than the fhMetS subjects with a normal body mass. The atherogenic index (TG/HDL-C ratio) was significantly higher in the overweight and obese fhMetS participants compared to the fhMetS participants with normal body mass (p < 0.002) (Table 3). The fhMetS subjects had significantly lower mean fasting glucose values (4.51 mmol/L vs. 4.81 mmol/L, p < 0.042) and higher fasting insulin values, although this difference was not significant (Table 2). A significant positive correlation between the fasting glucose and fasting insulin concentrations was detected in the fhMetS group (r = 0.28, p < 0.015) but not in the control group. HbA1c levels were within the normal range in both groups (p = 0.748). Significantly higher D-SBP, D-DBP and N- DBP (p < 0.035, p < 0.045, and p < 0.019) were present in the fhMetS group than in the control group (Table 4). The frequency of the daytime SBP values > 140 mmHg and DBP values >90 mmHg in the fhMetS group were 3.9% and 7.8% respectively, while the frequency of nighttime SBP values > 125 mmHg and DBP values >80 mmHg were 5.1% and 1.3% respectively.

**Table 1:** Clinical characteristics of the studied groups (medians and IQR)

	**fhMetS (n = 78)**	**Control group (n = 12)**	**p**
Age (years)	35.0 (31.0–39.0 )	32.5 (27.0–37.0 )	0.256
Body mass (kg)	75.5 (59.0–93.0)	69.5 (58.0–81.0)	0.267
WC (cm)	84.5 (70.0–97.5)	77.9 ( 67.0–88.1)	0.102
BMI (kg/m^2^)	25.3 (21.9–28.7 )	23.6 (22.5–26.0)	0.499
WHR	0.9 (0.8–0.9)	0.8 (0.8–0.9)	0.365
Creatinine (mmol/L)	79.56 (53.04–106.08)	53.04 (61.88–97.24)	0.681
eGFR (ml/min)	84.4 (74.9–92.7)	82.3 (71.7–89.9)	0.420
Uric acid (umol/L)	261.71 (220.0–339.0)	261.72 (237.92–285.5)	0.879
AST (U/L)	19.0 (16.0–24.0)	17.1(13.0–22.0)	0.248
ALT (U/L)	22.0 (15.0–32.0)	13.5 (10.0–22.5)	0.118
ESR (mm)	7.0 (4.0–13.5)	8.0 (6.0–16.0)	0.462
WBC (G/L)	6.1 (5.1–7.2)	6.9 (5.2–9.7)	0.310
hsCRP (mg/L)	1.3 (0.8–2.5)	1.1 (0.4–5.8)	0.704

WC- waist circumference, BMI- body mass index, WHR- waist/hip ratio, GFR- glomerular filtration rate, AST-aspartate transaminase, ALT- alanine aminotransferase, ESR erytrocyte sedimentation rate, WBC- white blood cell count, hsCRP- high-sensitivity C-reactive protein.

**Table 2:** Parameters of lipid and carbohydrate metabolism in studied groups (medians and IQR)

	**fhMetS (n = 78)**	**Control group (n = 12)**	**P**
TC (mmol/L)	5.46 (2.21–6.08)	4.69 (1.27–5.55)	< 0.030
LDL-C (mmol/L)	3.28 (2.68–3.95)	2.90 (2.39–3.07)	< 0.032
HDL-C (mmol/L)	1.46 (1.17–1.51)	1.51 (1.16–1.65)	0.976
nonHDL-C (mmol/L)	3.74 (3.30–4.63)	3.25 (2.64–3.70)	< 0.016
TG (mmol/L)	1.03 (0.75–1.63)	0.77 (0.61–1.31)	0.339
Fasting glucose (mmol/L)	4.51 (4.29–4.73)	4.81 (4.54–5.01)	< 0.042
Fasting insulin (μU/ml)	6.2 (4.3–8.3)	5.9 (5.1–8.2)	0.871
HOMA-IR	1.24 (0.82–1.83)	1.27 (1.01–1.94)	0.748
HbA1c (%)	5.5 (5.3–5.7)	5.5 (5.3–5.5)	0.268

TC- total cholesterol, LDL-C low- density lipoprotein cholesterol, HDL-C- high- density lipoprotein cholesterol, TG- triglyceride, HbA1c-glycated hemoglobin A1c.

**Table 3:** Lipid parameters (mmol/L) in fhMetS depending on BMI; (medians and IQR)

**mmol/L**	**1. Normal (n =37)**	**2. Overweight (n = 26)**	**3. Obese (n = 15)**	**P 1vs2**	**P 1vs3**	**P 2vs3**
TC	5.41 (4.77–6.02)	5.54 (4.81–5.98)	5.07 (4.21–6.45)	0.798	0.681	0.749
LDL-C	3.20 (2.61–3.67)	3.46 (2.76–4.16)	3.25 (2.29–4.91)	0.199	0.614	0.813
HDL-C	1.69 (1.44–2.03)	1.25 (1.14–1.46)	1.12 (0.88–1.35)	0.0007	0.0001	0.041
nonHDL-C	3.55 (2.98–4.16)	4.29 (3.38–4.63)	3.90 (3.33–5.54)	0.094	0.154	0.922
TG	0.82 (0.52–1.13)	1.21 (0.92–1.80)	1.64 (0.75–2.59)	0.0007	0.012	0.627
TG/HDL ratio	1.18 (0.55–1.59)	2.23 (1.63–3.40)	3.35 (1.27–6.53)	0.0001	0.002	0.294

Normal -BMI < 25 kg/m^2^, Overweight- BMI ≥ 25 kg/m^2^, Obese- BMI ≥ 30 kg/m^2^.

**Table 4:** The characteristics of study groups according to ABPM values (medians and IQR)

**mmHg**	**fhMetS (n = 78)**	**Control group (n = 12)**	**p**
D-SBP	121.5 (112.5–129.5)	113.3 (107.5–121.5)	<0.035
D-DBP	79.0 (74.0–83.5)	74.5 (68.5–78.0)	<0.045
N-SBP	105.0 (97.0–113.0)	101.0 (93.5–106.0)	0.244
N-DBP	64.0 (60.0–70.0)	59.5 (53.5–65.0)	<0.019

D-SBP- daytime systolic blood pressure, D-DBP- daytime diastolic blood pressure, N-SBP- nighttime systolic blood pressure, N-DBP- nighttime diastolic blood pressure.

## Discussion

Based on the comparison of young healthy adults who were children of parents with confirmed MetS, and age- and sex-matched healthy controls without a family history of MetS in the present study, we detected differences in some of the MetS components. Furthermore, more than half of the adults with a family history of MetS had abnormal body mass, and 1 in 5 were obese. Owing to the similarity in nutritional and physical activity habits between the groups, the high prevalence of abnormal body mass in the adults with a family history of MetS may reflect a predisposition to increased body weight in these individuals. A number of epidemiological studies have shown a relationship between excess body mass (overweight and obesity) and all-cause and cardiovascular mortality [18,19]. Moreover, adipose tissue, especially visceral adipose tissue, is related to abnormal lipid metabolism and induces insulin resistance in tissues. Atherogenic dyslipidemia is characterized by decreased HDL cholesterol concentrations, hypertriglyceridemia, and increased concentrations of small dense LDL particles. A strong positive correlation was found between the risk of cardiovascular disease and both TC and LDL concentrations in healthy men and women as well as in patients with symptomatic CVD [20]. Importantly, our results showed that more than 60% of the subjects with a family history of MetS had elevated TC (≥4.94 mmol/L) and LDL-C (≥2.99 mmol/L) levels in addition to a greater frequency of high TG concentrations and lower HDL-C values. As with BMI, this is likely related to a family predisposition for abnormal lipid profiles, rather than differences in nutritional and physical activity habits. The 2012 European Guidelines for the prevention of cardiovascular diseases recommend the use of non-HDL-C as a parameter to quantify the amount of atherogenic lipoproteins containing apolipoprotein B, which allows the prediction of CVD risk to a similar extent or even more accurately than LDL-C [5,21]. The present study reported a significantly higher mean non-HDL cholesterol concentration in the group with a family history of MetS than in the control group (p < 0.016). Regarding insulin levels, previous studies have demonstrated that, relationships exist between adipose tissue, insulin, the progression of insulin resistance, and hyperinsulinemia, although these relationships have not yet been well defined. A significant relationship has been demonstrated between insulin concentration and the risk of cardiovascular death, independent of other established risk factors, in people without diabetes [22]. Although the fasting glucose and insulin values were normal in both groups in the present study, a significant positive correlation was found between glucose and insulin concentrations in the group with fhMetS (r= 0.28, p < 0.015). This finding may indicate greater insulin resistance in tissues. However, HOMA-IR did not exceed 2.5 in any of the participants, and there was no differences in HOMA-IR between the groups. Experimental and clinical studies have also shown a relationship between adipose tissue and progression to and maintenance of elevated blood pressure [23]. The National Health and Nutrition Examination Survey III study results demonstrated that the prevalence of arterial hypertension increases with increasing BMI [24]. A positive correlation has also been found between BMI, lipid disorders, arterial hypertension, and the progression of atherosclerotic plaques in young people. In our study the ABPM resulted in significantly higher mean values of SBP and DBP in the daytime and DBP at nighttime in the participants with a family history of MetS, although less than 8% of these participants had elevated blood pressure. Population studies indicate that a family history of MetS is a marker of a strong genetic predisposition for cardiometabolic complications [25,26]. The results of the WHO–MONICA study, conducted in the French population, suggest that MetS should be assessed as an independent risk factor for the early onset of cardiovascular disease [25]. In the National Heart, Lung, and Blood Institute Family Heart Study, a family history of diabetes, and arterial hypertension resulted in a predisposition for carbohydrate and lipid metabolic disorders, which was especially true for non-obese individuals [26]. Such disorders appeared at a young age [27]. Similar results were demonstrated in the present study, in which there was a tendency for the early development of metabolic disorders in young persons with fhMetS. Collectively, the results of the present study indicate that abnormal body mass and lipid disorders, including those of total cholesterol and LDL cholesterol, are the most frequent metabolic disorders in young persons with a family history of MetS. This study has certain limitations, including a small sample size resulting from very rigorous inclusion and exclusion criteria intended to obtain homogenous groups. The parental history of MetS was confirmed in the fhMetS group and excluded in the control group.

However to the best of our knowledge, this is the first study documenting the typical metabolic alterations in MetS in healthy young adult children of parents with confirmed MetS. The results suggest that early screening for risk factors of MetS, such as overweight and hypercholesterolemia, should be conducted in this group. However, additional studies are required to confirm our findings.

## Conclusions

Owing to the high frequency of overweight/obesity and lipid disorders, which are considered MetS risk factors for cardiovascular disease, in the healthy young adults with a family history of MetS, this group should be considered at high risk and undergo regular screening.

## Competing interests

The authors declare that they have no competing interests.

## Authors’ contributions

AL, PP –acted as the principal investigators and contributed to study design and manuscript preparation, MK-B, KJ, AK, MC, AO-P, EM – assisted in individuals recruitment, data collection and discussions, ZL- performed the statistical analysis and participated in discussion, UD- contributed to laboratory analysis and participated in discussion. All authors read and approved the final manuscript.
